# Bimodal EEG-fNIRS in Neuroergonomics. Current Evidence and Prospects for Future Research

**DOI:** 10.3389/fnrgo.2022.934234

**Published:** 2022-08-12

**Authors:** Nicolas J. Bourguignon, Salvatore Lo Bue, Carlos Guerrero-Mosquera, Guillermo Borragán

**Affiliations:** ^1^Department of Life Sciences, Royal Military Academy of Belgium, Brussels, Belgium; ^2^GTM-Grup de Recerca en Technologies Mèdia, La Salle-Universitat Ramon Llull, Barcelona, Spain; ^3^Center for Research in Cognition and Neuroscience, Université Libre de Bruxelles, Brussels, Belgium

**Keywords:** multimodal brain imaging, electroencephalography, near-infrared spectroscopy, neuroergonomics, human-machine interfaces

## Abstract

Neuroergonomics focuses on the brain signatures and associated mental states underlying behavior to design human-machine interfaces enhancing performance in the cognitive and physical domains. Brain imaging techniques such as functional near-infrared spectroscopy (fNIRS) and electroencephalography (EEG) have been considered key methods for achieving this goal. Recent research stresses the value of combining EEG and fNIRS in improving these interface systems' mental state decoding abilities, but little is known about whether these improvements generalize over different paradigms and methodologies, nor about the potentialities for using these systems in the real world. We review 33 studies comparing mental state decoding accuracy between bimodal EEG-fNIRS and unimodal EEG and fNIRS in several subdomains of neuroergonomics. In light of these studies, we also consider the challenges of exploiting wearable versions of these systems in real-world contexts. Overall the studies reviewed suggest that bimodal EEG-fNIRS outperforms unimodal EEG or fNIRS despite major differences in their conceptual and methodological aspects. Much work however remains to be done to reach practical applications of bimodal EEG-fNIRS in naturalistic conditions. We consider these points to identify aspects of bimodal EEG-fNIRS research in which progress is expected or desired.

## Introduction

Neuroergonomics is the branch of human factors concerned with the live reading of brain functions underlying real-world human performance in the physical (e.g., walking/running, manipulating tools, driving) and cognitive domains (e.g., calculating, reasoning, communicating). Its main goal is to design human-machine interfaces to enable autonomous daily behaviors in people with movement or communication disorders or to ensure the efficiency and safety of complex, high-risk private or professional activities (e.g., flying, driving, operating machines, etc.). Achieving this goal not only requires understanding how brain activity represents performance-related mental states such as goals (e.g., intending to go left or right), feelings and emotions (e.g., stress or anxiety), or mental/physical effort (e.g., workload, exhaustion, etc.) but also on brain monitoring techniques that can decode these mental states quickly and accurately. Existing techniques with such potential include electroencephalography (EEG), which measures the brain's temporal unfolding of electrophysiological activity with a millisecond accuracy (Casson, 2019), and functional near-infrared spectroscopy (fNIRS), which enables precise localization of brain activity during performance (Ferrari and Quaresima, 2012; Vitorio et al., 2017; Quaresima and Ferrari, 2019; Zhu et al., 2020). A cousin of hemodynamic brain monitoring techniques such as positron emission tomography and fMRI, fNIRS uses the diffusion of infrared light emitted through the skull and refracted at various intensities depending on the concentration of (de)oxygenated hemoglobin (see Pinti et al., 2018a and Pan et al., 2019 for details). The possibility to acquire this signal through sensors placed on the scalp makes fNIRS significantly more portable than PET or fMRI, earning it increasing attention in many sectors of neuroergonomics and human factors design.

As an emergent discipline, neuroergonomics faces two important challenges. One is to miniaturize brain monitoring techniques in a way that allows for natural evolution in the real world without jeopardizing brain signal quality. Indeed, despite their higher flexibility of use compared with other techniques, both EEG and fNIRS remain sensitive to movement artifacts likely to contaminate brain signal. Another concerns the inherent temporal and spatial limitations of fNIRS and EEG, respectively. Like fMRI, fNIRS suffers an approximate 5-second delay between stimulus presentation and associated brain responses (Pinti et al., 2018a). This discrepancy poses a problem for studying the brain bases of performance in naturalistic conditions where events tend to happen quickly and unpredictably. Conversely, EEG's limited spatial resolution does not allow for accurate identification of brain regions at the source of task-relevant brain signal. Finally, fNIRS only provides partial information on the processes taking place within task-relevant brain regions, whereas electrode recordings permit finer-grained analyses of these processes in terms of timing and local or global neuronal interactions (Logothetis et al., 2001; Palva and Palva, 2012).

To overcome these challenges, recent efforts have been made to combine EEG and fNIRS into bimodal systems able the simultaneously record the hemodynamic and electrophysiological correlates of human performance in real time (Safaie et al., 2013; Tomita et al., 2014; Hong and Khan, 2017; von Luhmann and Muller, 2017; Hong et al., 2018). Indeed, besides improving the signal-to-noise ratio by representing brain activity in different formats (Sun et al., 2020), combined EEG and fNIRS signals have the potential to significantly compensate for each other's spatial and temporal limitations, thereby increasing the speed, precision, and richness of mental state decoding in various tasks of interest to human factors (Wallois et al., 2010, 2012; Nguyen et al., 2012, 2017; Tomita et al., 2014; Kaewkamnerdpong, 2016; Balconi et al., 2017; Pinti et al., 2018b; Dehais et al., 2019; Firooz and Setarhdan, 2019). They may ultimately provide motor-disabled individuals with the means to interact with their environment and optimize the detection of mental workload, drowsiness, or brain dysfunction in various high-risk situations.

Given the recency of bimodal EEG-fNIRS technologies, and despite several reports of their higher decoding accuracy compared to unimodal EEG and fNIRS, it is still unclear whether these improvements generalize over most processes typically investigated in human-machine interface research or whether they relate to particular domains of performance and not others (e.g., whether its decoding accuracy is higher for cognitive relative to physical performance). Another pending question is whether these improvements are stable despite variations across studies' methodology. To help elucidate these questions the present article reviews studies comparing the performance of bimodal EEG-fNIRS against unimodal EEG and unimodal fNIRS in performance-based mental state decoding in the context of key paradigms of neuroergonomics: motor imagery and execution for remote-controlled action, navigation for safe and efficient driving or flying, clinical diagnosis for efficient healthcare services, as well as cognitive and affective processing for detecting potentially harmful cognitive or affective states (e.g., stress, mental workload, drowsiness, etc.). We consider the relative value of bimodal vs. unimodal EEG and fNIRS in light of these studies' primary conceptual and methodological differences in order to identify areas in which improvement is expected or desired in the coming years. We also consider the state of progress achieved in building wearable versions of bimodal EEG-fNIRS equipment to be used in naturalistic conditions. These considerations provide the basis for recommendations as to how future neuroergonomics research should be carried out using multimodal brain imaging.

## Methods

**Figure 1:**
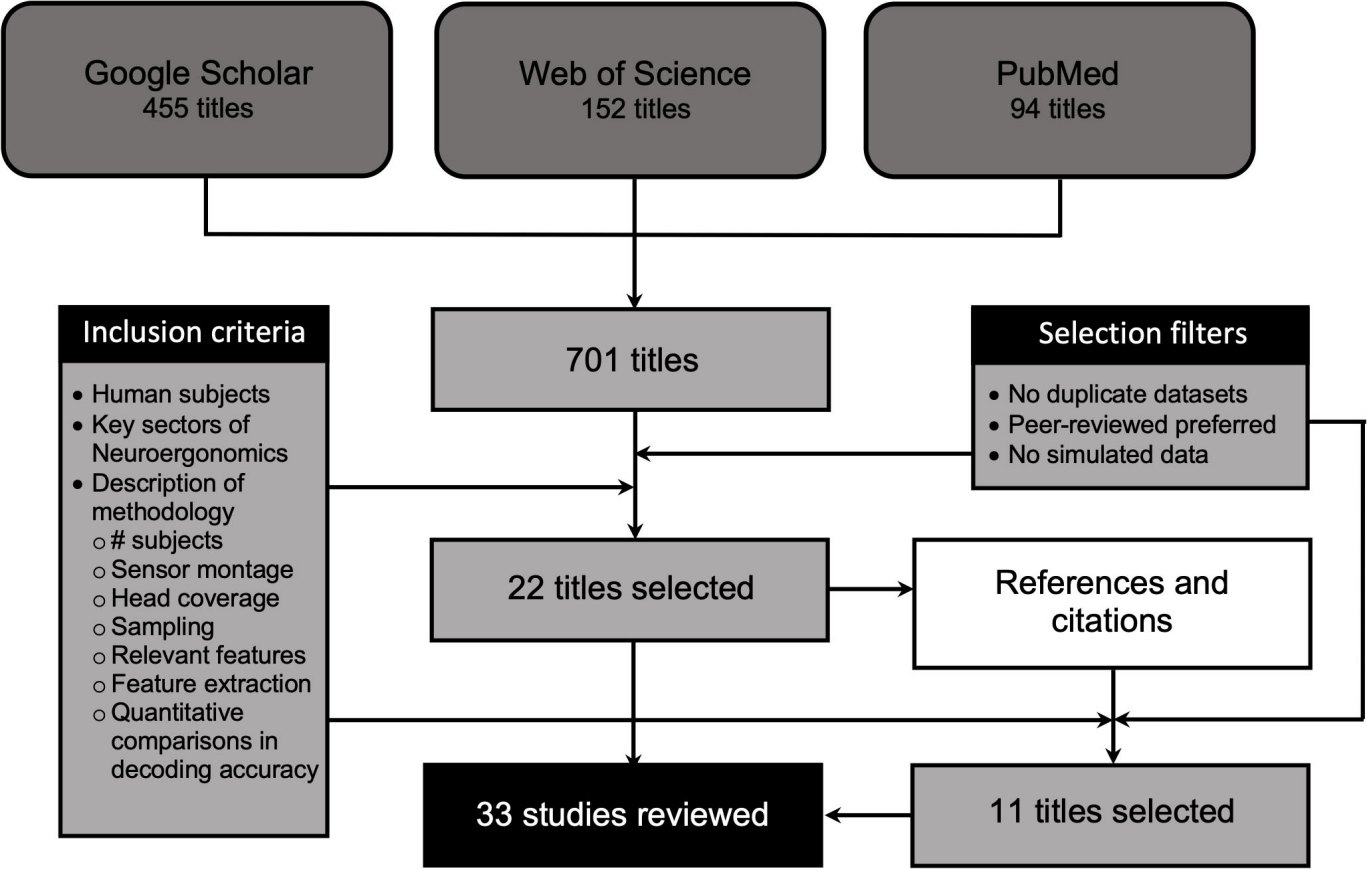
Flowchart of the literature search.

Given the recency and sparseness of mental state-decoding studies using bimodal EEG-fNIRS, we sought to maximize the chances of finding relevant studies by departing from the standards of systematic literature reviews (e.g., Moher et al., 2009, see Figure 1 for a graphical representation of the study selection process). This review should therefore not be regarded as systematic. The literature search was initiated in 2020 and terminated in 2021. A Google Scholar search was performed *via* the Publish or Perish search engine (Harzing, 2007, Publish or Perish, available https://harzing.com/resources/publish-or-perish) with the following keywords: “EEG-fNIRS” AND “simultaneous” OR “concurrent” AND “bimodal” OR “multimodal” OR “feature classification”, yielding a total of 455 relevant titles. Additional searches were performed using the same engine in PubMed and Web of Science using the keyword “EEG-fNIRS”, yielding 94 titles for PubMed and 154 titles for Web of Science. Prospective studies were reviewed based on the following criteria: (a) studies performed on human subjects, (b) research contributing to key sectors of neuroergonomics research, (c) description of key methodological points, specifically: (1) the number of subjects tested, (2) sensor montage and type of hardware used (i.e. custom-made vs. commercially distributed sensors), (3) head coverage specifying which parts of the brain were targeted, (4) the sampling parameters for EEG and fNIRS, (5) the EEG and fNIRS features of interest extracted and (6) the feature extraction method used (this information is provided in Table 1), (d) direct, quantitative comparisons of mental state classification accuracy between unimodal EEG, unimodal fNIRS and bimodal EEG-fNIRS.

**Table 1:** Target methodological details of the studies included in the review.

**Paradigm**	**References**	**# Subjects**	**Montage**	**Head coverage**	**Sampling parameters**	**Feature extracted**	**Feature classification method**
Motor imagery	Yin et al. (2015)	6	EEG: Neuroscan (Synamps2), 21 electrodes	Left right motor cortex	EEG: 1000Hz SR	EEG: EEG power, instantaneous amplitude, phase, and frequency	Extreme learning machine (ELMs)
			NIRS: ETG-4000 (Hitachi), 10 emitters, 8 detectors, 24 channels		NIRS: 696-830 nm WL, 10 Hz SR	NIRS: HbR, HbO, HbT and HbD	
	Koo et al. (2015)	6	EEG: gMOBIlab+ (gTec), 6 electrodes	Left right (pre)motor cortex	EEG: 256 Hz SR	EEG: alpha, beta, delta, and theta band power	Support Vector Machines (SVM)
			NIRS: Imagent (ISS Inc.), 8 emitters, 2 detectors		NIRS: 690-830 nm WL, 6.25 Hz SR	NIRS: HbO	
	Blokland et al. (2014)	8 patients	EEG: Porti (TMSi), 8 electrodes	Left/right (pre)motor cortex	EEG: 2048 Hz SR	EEG: ERD	L2 regularized linear logistic regression classifier
		12 controls	NIRS: Oxymon MK III (Artinis), 4 emitters, 2 detectors, 2 channels		NIRS: 764-858 nm WL, 250 Hz SR	NIRS: HbO, HbR	
	Leamy et al. (2011)	2	EEG: Active Two (Biosemi), 7 electrodes	Left/right motor cortex	EEG: 2,048 Hz SR	EEG: ERS/ERD	Linear Discrimination Analysis (LDA)
			NIRS: TechEn CW6 (TechEn), 3 sources, 3 detectors, 7 channels		NIRS: 690–830 nm WL, 25 Hz SR	NIRS: HbO and HbR	
	Fu et al. (2020)	6	EEG: Neuroscan (SynAmps2), 2 electrodes	Left/right motor cortex	EEG: 1,000 Hz SR	EEG: IA, IP, IF (single feature vector)	SVM
			NIRS: ETG-4,000 (Hitachi), 2 emitters, 2 detectors, 2 channels		NIRS: 695–830 nm WL, 10 Hz SR	NIRS: HbO, HbD (HbO/HbR) EEG-fNIRS: HbO+HbD+IA+IP+IF	
	Fazli et al. (2012)	14	EEG: BrainAmp (Brain Products), 37 electrodes	Frontal, motor, parietal cortex	EEG: 1,000 Hz SR	EEG: alpha and beta bands	LDA
			NIRS: NIRScout 8-16 (NIRx), 8 emitters, 16 detectors, 24 channels		NIRS: 760–850 nm WL, 6.25 Hz SR	NIRS: HbO, HbR	
	Saadati et al. (2020)	29	EEG: BrainAmp (Brain Products), 30 electrodes	Frontal, motor, visual cortex	EEG: 200 Hz SR	EEG: ERD/ERS	Deep Neural Networks (DNN)[3]
			NIRS: NIRScout (NIRx), 16 emitters, 16 detectors, 28 channels		NIRS: WL not given, 10 Hz SR	NIRS: HbO, HbR EEG-fNIRS: Fused ERD/ERS-HbO-HbR	
	Ge et al. (2017)	12	EEG: Neuroscan (SynAmps2), 64 electrodes	Left/right motor cortex	EEG: 1,000 Hz	EEG: Current density	SVM
			NIRS: LABNIRS (Shimadzu), 11 emitters, 11 detectors, 31 channels		NIRS: 780, 805, and 830 nm WL, 28 Hz WL	NIRS: Hurst Exponent	
	Verma et al. (2019)	9	EEG/NIRS: g.Nautilus fNIRS^*^ (gtec)	NIRS: Left/right motor cortex	EEG: 250 Hz SR	EEG: mu and beta bands	LDA
			EEG: 15 electrodes NIRS: 8 emitters, 2 detectors	EEG: Left/right (pre)motor and parietal cortex	NIRS: 760-850 nm WL, 250 Hz SR	NIRS: HbO, HbR	
	Chiarelli et al. (2018)	15	EEG: System Net 300 (Electrical Geodesics), 123 electrodes	NIRS: Left/right (pre)motor cortex	EEG: 256 Hz SR	EEG: ERD/ERS	DNN
			NIRS: Imagent (ISS Inc.), 16 sources, 2 detector	EEG: Full head	NIRS: 690–830 nm WL, 10 Hz SR	NIRS: HbO, HbR	
Motor execution	Blokland et al. (2014)	8 patients	EEG: Porti (TMSi), 8 electrodes	Left/right (pre)motor cortex	EEG: 2,048 Hz SR	EEG: ERD	L2 regularized linear logistic regression classifier
		12 controls	NIRS: Oxymon MK III (Artinis), 4 emitters, 2 detectors, 2 channels		NIRS: 764–858 nm WL, 250 Hz SR	NIRS: HbO, HbR	
	Leamy and Ward (2010)	2	EEG: Active Two (Biosemi), 7 electrodes	Left/right motor cortex	EEG: 2,048 Hz SR	EEG: ERS/ERD	LDA
			NIRS: TechEn CW6 (TechEn), 3 emitters, 3 detectors, 7 channels		NIRS: 690-830 nm WL, 25 Hz SR	NIRS: HbO, HbR	
	Li et al. (2017)	11	EEG: BrainAmp DC (Brain Products), 16 electrodes	Left/right motor cortex	EEG: 500 Hz SR	EEG: Discrete wavelet transform of EEG signal	SVM
			NIRS: NIRScout (NIRx), 12 sources, 12 detectors (34 channels)		NIRS: 760–850 nm WL, 7.81 Hz SR	NIRS: Stimulus-related initial HbO/HbR dip	
	Buccino et al. (2016)	15	EEG: microEEG (BioSignal Group), 21 channels	Left/right (pre)motor cortex	EEG: 250 Hz	EEG:ERP/ERS	LDA
			NIRS: NIRScout 8-16 (NIRx), 12 emitters, 12 detectors, 34 channels		NIRS: 760-850 nm WL, 10.42 Hz SR	NIRS: HbO, HbR	
	Fazli et al. (2012)	14	EEG: BrainAmp (Brain Products), 37 electrodes	Frontal, motor, parietal cortex	EEG: 1,000 Hz SR	EEG: alpha and beta bands	LDA
			NIRS: NIRScout 8-16 (NIRx), 8 emitters, 16 detectors, 24 channels		NIRS: 760–850 nm WL, 6.25 Hz SR	NIRS: HbO, HbR	
	Zhu et al. (2017)	3	EEG: BrainAmp (Brain Products), 4 electrodes	Left/right motor cortex	EEG: 550 Hz SR	EEG: Wavelet approximation coefficients	LDA
			NIRS: NIRx, 8 sources, 8 detectors, 20 channels		NIRS: 760–850 nm WL, 7.81 Hz SR	NIRS: HbO	SVM
	Al-Quraishi et al. (2021)	20	EEG/NIRS: MCScap (Medical Computer Systems)	EEG/NIRS: Frontal/central/parietal cortex	EEG: 256 Hz SR	EEG: ERD/ERS (alpha band)	SVM
			EEG: 19 electrodes NIRS: 16 emitters, 16 detectors, 48 NIRS channel		NIRS: 695–830 nm, 10 Hz SR	NIRS: HbO, HbR	
Navigation (real or simulated)	Dehais et al. (2019)	4	EEG: Enobio (Neuroelectrics), 23 electrodes	EEG: Full head	EEG: 500 Hz SR	EEG: spectral density based on alpha, beta, and theta frequency bands	LDA
			NIRS: NIRSport (NIRx), 7 emitters, 8 detectors, 12 channels.	NIRS: Frontal temporal cortex	NIRS: WL not given, 8.93 Hz SR	NIRS: wavelet coherence based on HbO	
	Ahn et al. (2016)	11	EEG: ActiveTwo (Biosemi), 64 electrodes	EEG: Full head	EEG: 512 Hz SR	EEG:Beta/alpha ratio	LDA (for EEG)
			NIRS: custom-built, 2 emitters, 8 detectors, 8 channels	NIRS: Frontal cortex	NIRS: 735–850 nm WL, 10 Hz SR	NIRS: HbO, HbR	
Cognitive processing	Morioka et al. (2014)	8	EEG: ActiveTwo (Biosemi), 64 electrodes	EEG: Full head	EEG: 256 Hz SR	EEG: Alpha desynchronization in visual cortex	Sparse logistic regression
			NIRS: FOIRE-3000 (Shimadzu Co.), 15 emitters, 15 detectors, 49 channels	NIRS: Parietal occipital cortex	NIRS: 780–805–830 nm WL, 4 Hz SR	NIRS: HbO	
	Saadati et al. (2020)	26	EEG: BrainAmp (Brain Products), 30 electrodes	Frontal, motor, visual cortex	EEG: 200 Hz SR	EEG: ERD/ERS	DNN (SVM)
			NIRS: NIRScout (NIRx), 16 emitters, 16 detectors, 16 channels		NIRS: WL not given, 10 Hz SR	NIRS: HbO, HbR	
	Shin et al. (2017)	10	EEG: BrainAmp (Brain Products), 22 electrodes	EEG: Full head	EEG: 1,000 Hz SR	EEG: alpha, beta, theta	LDA
			NIRS: NIRScout (NIRx), 5 sources, 3 detectors	NIRS: frontal cortex	NIRS: WL not given, 12.5 Hz SR	NIRS: HbR, HbO	
	Aghajani et al. (2017)	17	EEG: microEEG (Bio-Signal), 19 electrodes	EEG: Full head	EEG: 259 Hz SR	EEG: Frequency band power, phase-locking value, frequency, left-right asymmetry of delta, theta, alpha, low beta, and high beta bands	SVM
			NIRS: NIRScout (NIRx), 19 emitters, 19 detectors, 19 channels	NIRS: Frontal cortex	NIRS: 760-850 nm WL, 8.93 Hz SR	NIRS: HbO, HbR (amplitude, slope, standard deviation, skewness, and kurtosis)	
	Coffey et al. (2012)	12	EEG: Guger Technologies, 8 electrodes	Frontal cortex	EEG: 256 Hz SR	EEG: band power within 2–25 Hz range	
			NIRS: Oxy-monMkIII (Artinis), 3 emitters, 3 detectors, 3 channels		NIRS: 766–860 nm WL, SR unknown	NIRS: HbO, HbR, HbT	
	Herff et al. (2015)	10	EEG: ANT, 3 electrodes	EEG: Fz, Cz, Pz	EEG: 256 Hz SR	EEG: 4–25 Hz band power	LDA
			NIRS: Imagent, 28 sources, 15 detectors (channels not defined)	NIRS: Frontal cortex	NIRS: 690–830 nm WL, 19.5 Hz SR	NIRS: HbO	
	Ge et al. (2019)	16	EEG: Neuroscan Synamps, 64 electrodes	Full head	EEG: 1,000 Hz SR	EEG: ERP Current Source Density (sLORETA)	Complex Brain Network
			NIRS: LABNIRS, 16 emitters, 16 detectors, 48 channels		NIRS: 780, 805 and 830 nm WL, 0.01–0.1 Hz SR	NIRS: HbO, HbR, HbT	
	Sereshkeh et al. (2019)	11	EEG: BrainAmp (Brain Products), 32 electrodes	EEG: Full head	EEG: 1,000 Hz SR	EEG: Discrete wavelet transform coefficient	LDA
			NIRS: ETG-4000 (Hitachi), 16 emitters, 14 detectors, 44 channels	NIRS: Frontal, temporal, parietal cortex	NIRS: 695–830 nm WL, 10 Hz SR	NIRS: HbO	
Affective/ emotional processing	Sun et al. (2020)	12	EEG: EMOTIV Epoc, 14 electrodes	EEG: Full head	EEG: 128 Hz SR	EEG: Power spectral density based on theta, slow alpha, alpha, and beta frequency bands	SVM
			NIRS: 1100 W (fnirdevices), 4 optodes 2 light wavelengths channels and one ambient channel per optode	NIRS: Frontal cortex	NIRS: WL not given, 4 Hz SR	NIRS: HbO, HbR, HbT (HbO+HbR)	
	Al-Shargie et al. (2016)	22	EEG: BrainMaster 24E, 7 electrodes	Frontal cortex	EEG: 256 Hz SR	EEG: Mean power alpha and beta frequency bands	SVM
			NIRS: OT-R40 (Hitachi), 8 emitters, 8 detectors, 23 channels		NIRS: 695-830 nm WL, 10 Hz SR	NIRS: HbO	
Clinical diagnosis	Abtahi et al. (2020)	9 patients	EEG: g.USBAMP (gtec), 13 electrodes	Left/Right motor cortex	EEG: 256 Hz SR	EEG: Alpha, theta and theta frequency bands	SVM
		9 controls	NIRS: NIRScout (NIRx), 8 emitters, 8 detectors, 16 channels		NIRS: 760–850 nm WL, 7.81 Hz SR	NIRS: HbO, HbR	
	Sirpal et al. (2019)	40 patients	EEG: Neuroscan Synamps 2TM, 19 electrodes	Full head	EEG: 500 Hz SR	EEG: Interictal epileptiform discharges	DNN
			NIRS: Imagent (ISS), 64 emitters, 16 detectors, channels unknown		NIRS: 690–830 nm WL, 19.5 Hz SR	NIRS: HbO, HbR	
	Cicalese et al. (2020)	Healthy: 8	EEG: BrainAmp DC (Brain Products), 32 electrodes	Frontal, parietal cortex	EEG: 500 Hz SR	EEG: Power spectrum density of delta, theta, low alpha, high alpha, beta, and gamma frequency bands	LDA
		Mild AD: 6	NIRS: NIRScout (NIRx), 16 sources, 16 detectors, 46 channels		NIRS: 760-850 nm WL, 3.91 Hz SR	NIRS: HbO, HbR	
		Severe AD: 7				Feature optimization with Pearson correlation coefficient based feature selection on EEG and NIRS	
		Mild CI: 8					
	Othman et al. (2020)	Patients: 9	EEG/NIRS: StarStim NIRS-EEG (Artinis)^*^	Frontal, motor, and parietal cortex	EEG: 500 Hz SR	EEG: 1-12 Hz band power	Adaptive mixture-independent k-nearest neighbor
		Controls: 14	EEG: 8 electrodes NIRS: 8 emitters, 2 detectors, 8 channels		NIRS: 760-850 nm WL, 50 Hz SR	NIRS: HbO	
	Güven et al. (2020)	Patients: 23	EEG: MP150 (Biopac System), 4 electrodes	EEG: Fz, Cz, Pz, Oz	EEG: 2500 Hz, SR	EEG: P300	SVM
		Controls: 21	NIRS: Imager 1100 (fNIR devices), 5 emitters, 2 detectors	NIRS: Frontal cortex	NIRS: 730–850 nm WL, 2 Hz	NIRS: HbO	Multilayer Perception Neural Network (MLP)
							Naïve Bayes (NB)
Other	Shin et al. (2018a,b)	18	EEG: ActiveTwo (Biosemi), 21 electrodes	Frontal cortex	EEG: 2,048 Hz SR	EEG: Filter-bank common spatial pattern on theta, alpha, and beta bands, one-vs.-one	LDA
			NIRS: LIGHTNIRS (Shimadzu), 6 emitters, 6 detectors, 16 NIRS channels		NIRS: WL not given, 13.3 Hz SR	NIRS: HbO, HbR, one vs. one (read about it) EEG and NIRS classifiers were then combined to construct new feature vectors for the meta-classifier.	
	Putze et al. (2014)	12	EEG: (asalab) ANT, 12 electrodes	Auditory, visual cortex	EEG: 256 Hz SR	EEG: Power spectral density and ERP waveform	LDA (HbO, HbR, and POW)
			NIRS: Imagent (ISS), 32 emitters, 16 detectors, number of channels unknown		NIRS: 690-830 nm WL, 110 MHz SR	NIRS: HbO, HbR	SVM (ERPs)

*Refer to the core text for further detail and the supplementary document for full bibliographic details*.

Complementing these criteria were the following three selection filters: First, when two articles reported results from the same dataset (e.g., Fazli et al., 2012; Lee et al., 2014; Ahn et al., 2016; Nguyen et al., 2017; Shin et al., 2017) only one of these articles was retained to avoid redundancy or selection biases. Second, when the same dataset was reported in peer-reviewed articles and conference proceedings (Fazli et al., 2012; Lee et al., 2014) only the peer-reviewed articles were retained. Finally, studies performed on simulated datasets (e.g., Croce et al., 2017) were excluded. As most of the research using bimodal EEG-fNIRS is still quite recent, the year of publication was not used as an inclusion/exclusion criterion. In total 22 articles were selected for inclusion in the review. In addition to the database search described above, each of the 22 articles found was then screened for its reference section and recent contributions citing it. Eleven additional articles were found that met the above criteria. A total of 33 research articles were therefore included in the review.

To better capture and discuss the conceptual, empirical, and methodological implications of the studies reviewed here, the following information was systematically extracted for each of them (see Table 1 for detail):

1. *Paradigm*. Studies were grouped into seven categories based on their paradigms. These categories were created in a way that best encapsulates the main domains of performance investigated in the literature. These include: (a) motor imagery (10 studies), (b) motor execution (seven studies), (c) navigation (simulated and/or real-world, 2 studies), (d) cognitive processing (working memory, word generation, mental arithmetic, spatial attention, 8 studies), (e) affective/emotional processing (2 studies) and (f) clinical (5 studies). An additional category ‘other' was created to include one study focused on discriminating mental states related to different tasks belonging to one of the six previous categories as well as one study aimed at discriminating between auditory and visual processing (Putze et al., 2014). It must be noted that some of the studies included in the review involved more than one task that could be assigned to different categories. Furthermore, one study involving clinical subjects differed from the other clinical studies in being focused on mental state decoding during motor imagery in patients and healthy controls (i.e. was not run for diagnostic purposes, cf. Blokland et al., 2014). Accordingly, this study was assigned to the motor imagery category, ensuring that the results of the two groups were given separately.
Naturally, this categorization can to a large extent be regarded as arbitrary. Alternative categorizations might help place greater emphasis on other equally relevant, possibly more specific aspects of performance such as mental workload, engagement, cognitive fatigue, or drowsiness. Similarly, the studies reviewed might be grouped in a way that more explicitly underlines the distinction between “physical” and “cognitive” performance. Several reasons however motivate the categorization proposed here. First, many of these alternative dimensions of performance are embedded in the context of more general activities in a way that makes it difficult to distinguish them. EEG-fNIRS studies on mental fatigue or drowsiness, for instance, have typically been carried out in the context of navigation tasks. Similarly, even the most elementary tasks of physical performance entail some level of cognitive processing (e.g., deciding to move left or right), while classical cognitive tasks such as working memory span or arithmetic are typically translated into physical responses. It is therefore not obvious where the cognitive dimension of performance ends and the physical one begins. Finally, the way the studies are categorized does not for the present purposes significantly affect the primary finding of the present review, as will be shown in the next section. For these reasons, we elected to consider these dimensions in more detail when commenting on the results of our analysis.
2. *Study parameters*, including the number of subjects, specifics on the fNIRS and EEG sensor setup, EEG and fNIRS sampling parameters (EEG: sampling rate, fNIRS: wavelength and sampling rate), EEG and fNIRS features of interest, as well as a detailed description of data preprocessing (including both feature extraction and feature classification).
3. *Mental state classification methods*, with a special focus on the machine learning algorithms used for decoding performance-related mental states.

## Description of Studies

The penultimate column in Table 2 provides the percentage mental state decoding accuracy between unimodal EEG, unimodal fNIRS, and bimodal EEG-fNIRS for every study reviewed. The reader is also referred to Table 1 for further methodological information on each study, to be discussed in more detail below. Despite substantial differences between the studies' paradigms and methodology results reveal consistently better classification accuracy for bimodal EEG-fNIRS compared to unimodal EEG or fNIRS. In the following we consider in detail the primary methodological differences between studies that are likely to affect bimodal EEG-fNIRS mental state classification accuracy, focusing on their paradigms, sensor layout, and signal pre-processing as well as EEG and fNIRS feature extraction, integration and classification methods for mental state decoding. We then examine the studies' level of ecological validity, which should help consider the main challenges and existing solutions for implementing bimodal EEG-fNIRS experiments outside the laboratory.

**Table 2:** Articles included in the review.

**Paradigm**	**References**	**Classification of interest**	**Decoding, classification accuracy measurement**	**Decoding accuracy (%)**	Source^*^
Motor imagery	Yin et al. (2015)	Clenching speed/force	EEG	88	T1/2/3
			fNIRS	76	
			EEG-fNIRS	**89**	
	Koo et al. (2015)	EEG: left/right grasping	EEG (classification accuracy)	90	T1
		NIRS: Motor imagery detection	fNIRS (detection accuracy)	98	
			EEG-fNIRS (True positive rate)	88	
	Blokland et al. (2014)	Finger tapping vs. Rest	*Patients—Imagined movements*		T1/2
			EEG	63	
			fNIRS (HbO+HbR)	65	
			EEG-fNIRS	**70**	
			*Controls - Imagined movements*		
			EEG	77	
			fNIRS	59	
			EEG-fNIRS	**79**	
	Leamy et al. (2011)	Ball squeezing	EEG[1]	53	T2
			fNIRS	56	
			EEG-fNIRS	**62**	
	Fu et al. (2020)	Clenching force/speed	*Trained and nontrained trials*		T3
			EEG (IA-IP-IF)[2]	72	
			fNIRS (HbO-HbR)	64	
			EEG-fNIRS (HbO-HbD and IA-IP-IF)	**74**	
	Fazli et al. (2012)	Left-right hand gripping	*Motor imagery*		
			EEG	78.2	T1
			fNIRS (HbO)	71.7	
			fNIRS (HbR)	65	
			EEG-HbO	**83.2**	
			EEG-HbR	**80.6**	
			EEG-HbO/R	**83.1**	
	Saadati et al. (2020)	Left/right hand movement	EEG	73	T6
			fNIRS (HbO+HbR)	83	
			EEG-fNIRS	**91**	
	Ge et al. (2017)	Left/right hand movement	EEG	74.7	T1
			fNIRS	56.8	
			EEG-fNIRS	**81.2**	
	Verma et al. (2019)	Left/right hand grasping	EEG	70	T1
			fNIRS (HbO)	71.2	
			fNIRS (HbR)	71.6	
			EEG-fNIRS (HbO)	**76.2**	
			EEG-fNIRS (HbR)	**78.7**	
			EEG-fNIRS (HbO+HbR)	**80**	
	Chiarelli et al. (2018)	Left/right hand squeezing	EEG	73.38	P8
			fNIRS	71.92	
			EEG-fNIRS	**83.28**	
Motor execution	Blokland et al. (2014)	Finger tapping vs. Rest	*Patients - Attempted movements*		T1/2
			EEG	73	
			fNIRS (HbO+HbR)	70	
			EEG-fNIRS	**79**	
			*Controls - Executed movement*		
			EEG	87	
			fNIRS (HbO+HbR)	77	
			EEG-fNIRS	**87**	
	Leamy and Ward (2010)	Active/Resting state	fEEG	79	T3
			fNIRS	75	
			EEG-fNIRS	**81**	
	Li et al. (2017)	Left/right hand movement	EEG	85.64	T2
			NIRS	85.55	
			EEG-fNIRS	**91.02**	
	Buccino et al. (2016)	Active/Rest	*Rest-task*		T1/2
		Left/right hand or arm movement	EEG	85.2	
			fNIRS [3]	69	
			fNIRS (+Slope indicator, SI)	92.4	
			EEG-fNIRS	86.2	
			EEG-fNIRS(+SI)	**94.2**	
			*Right-left response*		
			EEG	62.2	
			fNIRS	63.1	
			fNIRS (+SI)	70	
			EEG-fNIRS	67.1	
			EEG-fNIRS (+SI)	**72.2**	
	Fazli et al. (2012)	Left-right hand gripping	EEG	90.8	T1
			fNIRS HbO	71.1	
			fNIRS HbR	73.3	
			EEG-fNIRS/HbO	**92.6**	
			EEG-fNIRS/HbR	**93.2**	
			EEG-HbO/R	87.4	
	Zhu et al. (2017)	Left-right hand grasping	*LDA*		T1
			EEG	80.17	
			fNIRS	75.75	
			EEG-fNIRS	**83.33**	
			*SVM*		
			EEG	79.75	
			fNIRS	74.67	
			EEG-fNIRS	**84**	
	Al-Quraishi et al. (2021)	Ankle movement	EEG[4]	89.39	T5
			fNIRS	85.61	
			EEG-fNIRS	92.13	
Navigation (real or simulated)	Dehais et al. (2019)	High/low cognitive fatigue	*Simulated flight*		P547
			EEG	86.7	
			NIRS	81.5	
			EEG-fNIRS	**87.2**	
			*Real flight*		
			EEG	86.4	
			fNIRS	83.2	
			EEG-fNIRS	**87.6**	
	Ahn et al. (2016)	Sleep-deprived/Well-rested driving	EEG[5]	59.7	T3
			fNIRS	66.8	
			EEG+fNIRS	**68.3**	
Cognitive processing	Morioka et al. (2014)	EEG: Spatial attention	EEG	71.4	P133
		NIRS: Cortical activity detection	EEG-fNIRS	**79.1**	
	Saadati et al. (2020)	N-back: 0-, 2- and 3-back	*N-back*		T3/6
		Discrimination/selection: target vs. nontarget	EEG	67	
		Word generation vs. rest	NIRS (HbO+HbR)	80	
			EEG-fNIRS	**87**	
			*Discrimination selection response task*		
			EEG	71	
			fNIRS (HbO+HbR)	84	
			EEG-fNIRS	**91**	
			*Word generation*		
			EEG	72	
			fNIRS	85	
			EEG-fNIRS	**92**	
	Shin et al. (2018a,b)	Mental arithmetic/word chain performance	*Mental arithmetic (offline)*		P8/9
			EEG	84.9	
			fNIRS	79.1	
			EEG-fNIRS	**90**	
			*Word chain (offline)*		
			EEG	78.7	
			fNIRS	77.4	
			EEG-fNIRS	**85.5**	
			*Mental arithmetic (pseudo-online)*		
			EEG	81.5	
			fNIRS	75	
			EEG-fNIRS	**85.8**	
			*Word chain (pseudo-online)*		
			EEG	73.2	
			fNIRS	74.3	
			EEG-fNIRS	**79.8**	
	Aghajani et al. (2017)	*n*-back	*3back v 2back v 1back v 0back v Rest*		T3
			EEG	78	
			fNIRS	56.2	
			EEG-fNIRS	**85.4**	
	Coffey et al. (2012)	0-back, 1-back, 2-back	EEG	73.34	T1[6]
			fNIRS	61.34	
			EEG-fNIRS	73.08	
	Herff et al. (2015)	Digit recall (5-level workload)	EEG	90	P7[7]
			fNIRS	71	
			EEG-fNIRS	**93**	
	Ge et al., 2019	Action observation and intention classification	EEG	68.6	T1
			fNIRS	52.7	
			EEG-fNIRS	**72.7**	
	Sereshkeh et al. (2019)	Imagined speech (yes/no, rest)	EEG	63.76	T1[8]
			fNIRS	63.64	
			EEG-fNIRS	**70.45**	
Affective/emotional processing	Sun et al. (2020)	Affective state	*Image-content stimuli*		T2/3
			EEG	63	
			fNIRS	62	
			EEG-fNIRS	**75**	
			*Video content stimuli*		
			EEG-fNIRS	62	
			fNIRS	72	
			EEG	**80**	
	Al-Shargie et al. (2016)	Stress level	EEG	91.7	P13
			fNIRS	84.1	
			EEG-fNIRS	**95.1**	
Clinical diagnosis	Abtahi et al. (2020)	Healthy/Parkinson	*Patients vs. neurotypical controls[9]*		P6
			EEG	92.79	
			fNIRS	81.23	
			EEG-fNIRS	92.27	
			EEG-fNIRS-MoCap-WearUp flex sensors	**93.4**	
	Sirpal et al. (2019)	Seizure identification	EEG	97.6	T5[4]
			fNIRS	97	
			EEG-fNIRS	**98.3**	
	Cicalese et al. (2020)	Healthy/Alzheimer/Cognitive impairment	EEG	65.52	T2/4[4]
			fNIRS	58.62	
			EEG-fNIRS	**79.31**	
	Othman et al. (2020)	Unresponsive/low-responsive ICU patients vs. controls	*Unresponsive patients vs. controls*		T2
		ICU patients with vs. without recovery of consciousness	EEG	89	
			EEG-fNIRS	**97**	
			*Consciousness recovery vs. non-recovery*		
			EEG	82	
			EEG-fNIRS	**1**	
	Güven et al. (2020)	ADHD patients vs. controls (oddball detection)	*SVM*		T4^*^
			EEG	79.54	
			fNIRS	70.45	
			EEG-fNIRS	**86.36**	
			*MLP*		
			EEG	81.81	
			fNIRS	72.72	
			EEG-fNIRS	**86.36**	
			*NB*		
			EEG	79.54	
			fNIRS	77.27	
			EEG-fNIRS	**93.18**	
Other	Shin et al. (2018a,b)	Mental arithmetic, motor imagery, idle state	EEG	76.1	P5
			fNIRS	64.1	
			EEG-fNIRS	**82.2**	
			Subject-dependent classification		
	Putze et al. (2014)	Visual/auditory processing	EEG (POW)	83.5	
			EEG (ERP)	86.9	
			fNIRS (HbO)	75.8	
			fNIRS (HbR)	70.9	
			EEG-fNIRS	**94.7**	
			Subject-independent classification		
			EEG (POW)	71.8	T3
			EEG (ERP)	81.7	
			fNIRS (HbO)	66	
			fNIRS (HbR)	63.5	
			EEG-fNIRS	**88.6**	

*Please refer to Table 1 for further methodological details for each of these studies. Remarks: [1] Scores averaged over subjects, [2] IA = instantaneous amplitude, IP = instantaneous phase, IF = instantaneous frequency, [3] This study compared classification performance with or without the inclusion of a slope indicator (SI) as part of the classification algorithm for the NIRS signal. For reasons of clarity we distinguish values obtained with or without SI, [4] Only accuracy values are given, please refer to the articles for further classification values (e.g. precision, sensitivity, specificity, recall, etc.), [5] Classification values with additional information from electrocardiographic and electrooculographic features are omitted, [6] values averaged over subjects, [7] 1 vs. 5-back contrast only, [8] Results for all test blocks, [9] highest accuracy scores*.

* *This column provides information as to where in the original papers the results reported in the present review can be found: T: Table, P: Page. Bold values represent the highest decoding accuracy scores reported in each study*.

### Paradigms

**Figure 2:**
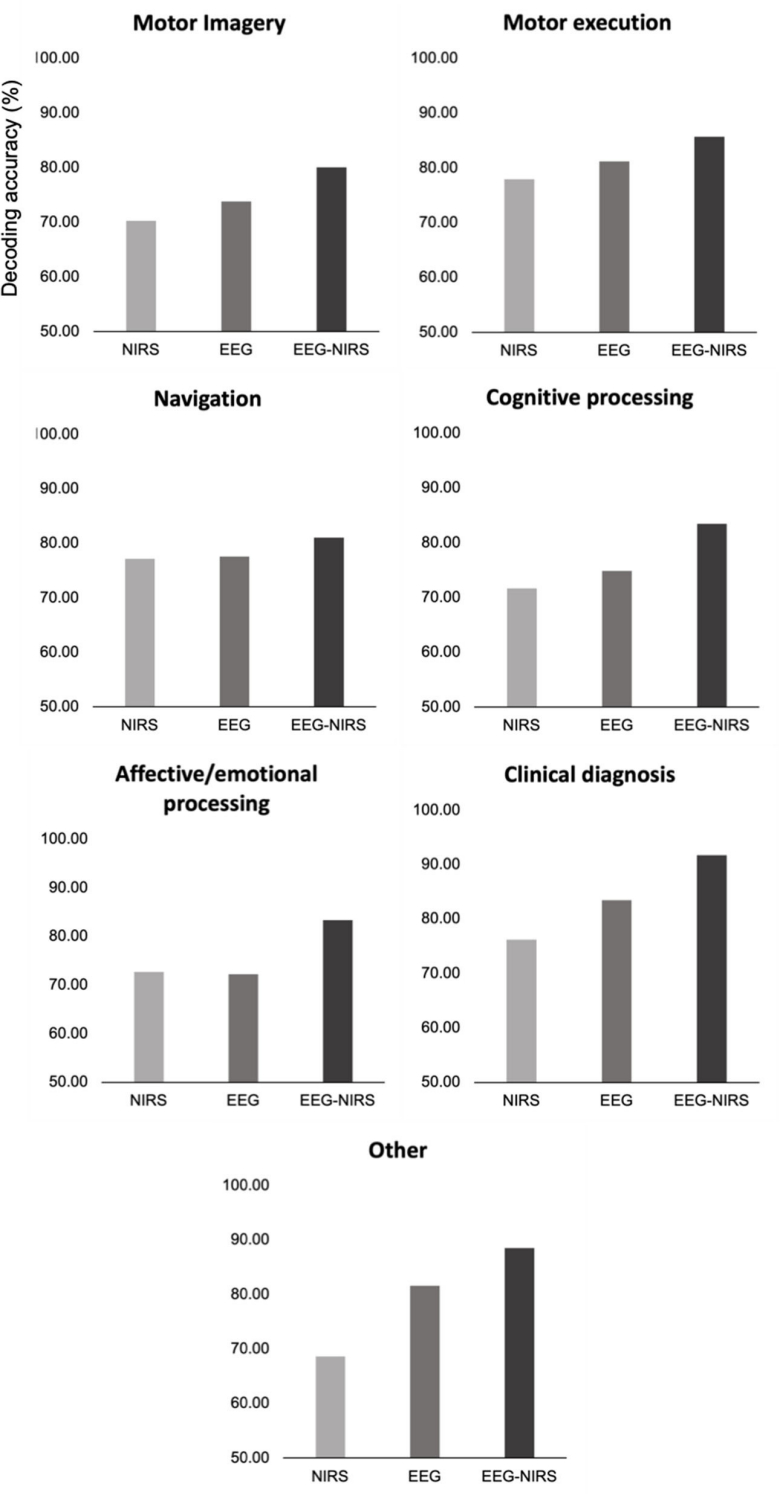
Differences in decoding accuracy between unimodal NIRS, unimodal EEG, and bimodal EEG-fNIRS across the seven paradigms considered. Scores were obtained by averaging percent decoding accuracy scores reported for each method by the studies reviewed in each category (see Tables 1, 2 for detail). When more than one possible score was obtained for the same individual method (e.g., when comparing different decoding algorithms), the highest score was systematically chosen. Note that these graphs are displayed for visual presentation and not statistical inference.

As shown in Figure 2, bimodal EEG-fNIRS yields consistent improvements in decoding accuracy compared to unimodal EEG and NIRS regardless of paradigm (we note that Figure 2 is provided only for visual observation and does not represent any statistical inference due to the relatively small number of studies included in each category). This section is devoted to commenting on some of the key aspects of the studies carried out within each of these paradigm categories.

Most studies were performed within research on brain-computer interface technologies (BCI) seeking to equip motor-disabled individuals with the means to communicate and interact with their environment (Allison et al., 2007; Ahn and Jun, 2018). Unsurprisingly, thus, many studies used tasks of motor imagery (11 studies) and motor execution (seven studies). Results show that bimodal EEG-fNIRS outperforms unimodal EEG and fNIRS in both cases, but bimodal classification appears more accurate in motor execution (~85%) compared to motor imagery tasks (~80%). This discrepancy could in part be explained by differences in the brain signatures associated with executed vs. imagined movements. Indeed, the hemodynamic signal appears more diffuse and weaker during motor imagery compared to motor execution, with limited involvement of primary motor regions (Deiber et al., 1998; Dechent et al., 2004). EEG studies comparing motor imagery vs. executed movements nevertheless reveal overall similar electrophysiological signatures in the motor regions between the two conditions, though classification in these regions was still highest during executed movements (Neuper et al., 2005). These studies also report significantly different activation profiles in the motor region depending on whether subjects are asked to imagine themselves or another person performing a motor action (Neuper et al., 2005), which highlights the impact of task demands and task instructions in driving classification accuracy. Further progress might therefore be partly contingent on a deeper understanding of the differences between motor imagery and motor execution at both the hemodynamic and electrophysiological levels.

Interestingly, despite the focus on the usefulness of EEG-fNIRS technologies to motor-disabled individuals, only one study actually involved patients with motor disorders (tetraplegia, cf. Blokland et al., 2014). Bimodal EEG-fNIRS in patients yielded higher classification accuracy than unimodal EEG or fNIRS for both imagined movement (EEG-fNIRS: 70%, fNIRS: 65%, EEG: 63%) and attempted movements (EEG-fNIRS: 79%, fNIRS: 70%, EEG: 73%). This finding highlights the potential use of EEG-fNIRS as an implementable neuroprosthetic technology. Other studies involving clinical populations were aimed at discriminating patients with neurological conditions such as Parkinson's disease (Abtahi et al., 2020), ADHD (Güven et al., 2020), Alzheimer's disease (Cicalese et al., 2020), or acute brain injury (Othman et al., 2020) from neurotypical controls, mostly within tasks targeting the neurological condition of interest (motor control in PD, attentional control in ADHD, memory retrieval in AD). These studies mostly showed higher classification accuracy overall for bimodal EEG-fNIRS (~90%) compared to unimodal EEG (~83%) or unimodal fNIRS (~77%), illustrating the utility of EEG-fNIRS for diagnostic purposes. One interesting exception is the study by Abtahi et al. (2020) investigating the accuracy of discrimination between PD patients and neurotypical controls based on unimodal EEG, unimodal fNIRS, bimodal EEG-fNIRS and bimodal EEG-fNIRS information fused with data from motion capture (MoCap) and WearUp flex sensors measuring large and small range hand movements, respectively. This study showed that while bimodal EEG-fNIRS does not perform better than unimodal EEG in discriminating PD from controls (92.27% for fNIRS compared to 92.79% for EEG), data from MoCap and WearUp measurements added to EEG-fNIRS significantly improved discrimination accuracy (93.4%). This suggests that additional information related to particular neurological conditions can further complement information from EEG-fNIRS to improve diagnostic accuracy.

Given the implications of neuroergonomics research in matters of public safety, another relevant sector interest for bimodal EEG-fNIRS technologies is the measurement of cognitive fatigue and engagement during navigation (driving and flying, Ahn et al., 2016; Dehais et al., 2019). The neural correlates of cognitive fatigue have been an important area of neuroergonomics research using EEG (Lal and Craig, 2001; Wascher et al., 2014; Mu et al., 2017), and several studies have now been conducted on cognitive fatigue using fNIRS (Borragán et al., 2018, 2019; Tanveer et al., 2019). A widely cited study in this respect reported higher accuracy of EEG-fNIRS in discriminating driving under well-rested vs. sleep-deprived conditions (Ahn et al., 2016). However, these authors also combined EEG-fNIRS with electrocardiography (ECG), achieving even higher discrimination compared to bimodal EEG-fNIRS (76% against 68.3%). Another study by Dehais et al. (2019) showed that EEG-fNIRS could appropriately decode levels of mental fatigue in pilots during simulated and real flight conditions, with little to no accuracy differences between the two (87.2% in simulation vs. 87.6% in a light aircraft). Altogether these findings not only stress the benefits of using bimodal EEG-fNIRS in parallel with other relevant physiological features to improve classification performance but also open the possibility to equip modern navigation systems with neurophysiological tools apt to precisely detect harmful levels of fatigue in moderate- to high-risk navigation situations.

Another central concept handled in half of the studies included in the “cognitive” category is that of mental workload—defined as the amount of information to be retained in working memory for prospective action (Krueger, 1989; Wickens, 2008; Loeppke et al., 2009; Mizuno et al., 2011; Raslear et al., 2011; Sievertsen et al., 2016). Here again, these studies report that bimodal EEG-fNIRS performs better (~85%) than EEG (~77%) or fNIRS alone (~67%) in decoding mental workload. It must be noted however that cognitive load is a multiplex concept encompassing several distinct but highly interactive components (Cain, 2007; Wickens, 2008). Beyond factors related to stimulus or task information, other intrinsic factors such as stress or emotional state (Al-Shargie et al., 2016) are also susceptible to modulating mental workload in multiple ways, as reflected in both hemodynamic and electrophysiological responses. For instance, Al-Shargie et al. (2016) investigated the neurocognitive correlates of stress in a task designed to tax cognitive processing. Their results revealed superior accuracy in stress identification for the bimodal technique (95.1%) compared to unimodal EEG (91.7%) and unimodal fNIRS (84.1 %). Altogether these studies highlight the practical payoffs of bimodal EEG-fNIRS in revealing the multiple facets of mental workload.

### Methodology

**Figure 3:**
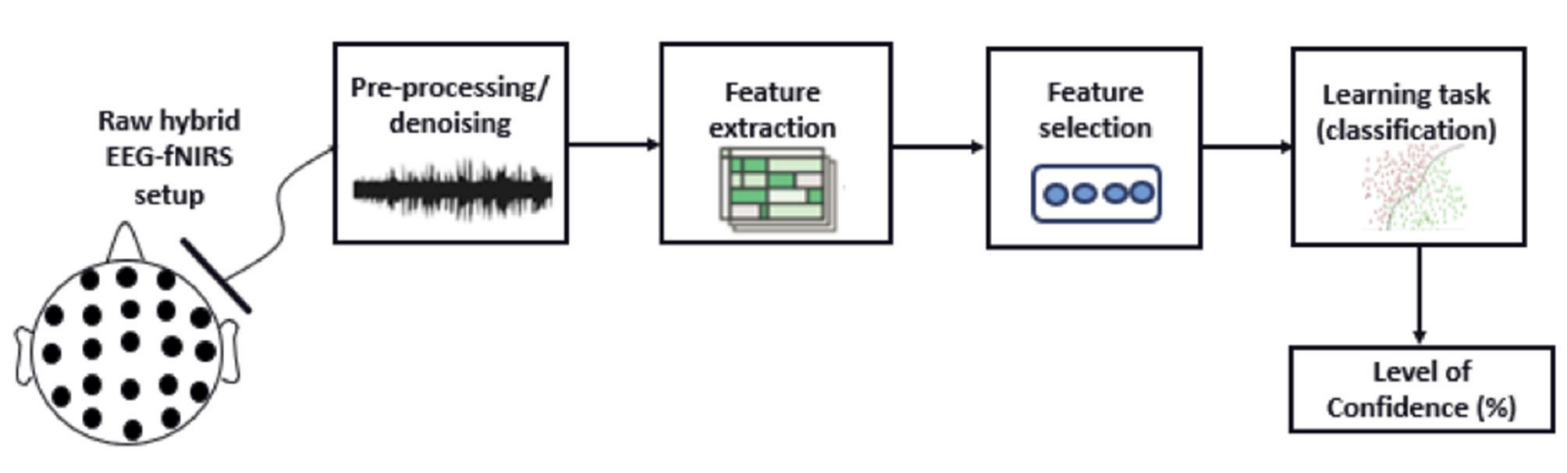
A standard acquisition and (pre-)processing pipeline using bimodal EEG-fNIRS systems for performance-related mental state classification.

Studies varied substantially in terms of their sensor setups, signal recording parameters, and mental state classification methods. To better present the implications of these methodological choices in bimodal EEG-fNIRS research we chose to describe them following a typical EEG-fNIRS recording-to-analysis pipeline represented in Figure 3 (for other examples see Hong and Khan, 2017). At each of the steps along this pipeline, we will describe the main options chosen in the studies reviewed, considering some of the advantages and challenges in the effort to achieve higher mental state decoding accuracy.

#### Sensor Setups, Scalp Coverage, and Signal Denoising

Studies varied substantially in their EEG and fNIRS sensor setup, notably regarding the number of sensors used and scalp coverage. In most cases, EEG and fNIRS channels were laid out on the same scalp locations under the assumption that they should record activity from the same underlying cortical sites. For instance, studies targeting hand-related motor movements mostly placed both EEG and fNIRS channels in the vicinity of the motor cortex (e.g., Leamy and Ward, 2010; Leamy et al., 2011; Blokland et al., 2014). Studies measuring mental workload focused on frontal regions typically associated with attention and working memory (Coffey et al., 2012; Herff et al., 2015), and studies investigating auditory vs. visual discrimination placed their EEG/fNIRS channels in regions corresponding to the primary auditory vs. visual cortex (Putze et al., 2014). Although these targeted placements make sense it should be remembered that EEG and fNIRS differ substantially in their spatial resolution. Indeed, despite a universal appeal to standardized layouts supposed to relate electrode locations to underlying cortical areas (e.g., the international 10–20 system), the notoriously poor spatial precision of traditional electrophysiological methods impede the mapping of EEG responses to functional anatomic patterns revealed by hemodynamic techniques (Laureys et al., 2009). In many cases, the scalp distribution of certain well-known EEG responses can be quite misleading as to their actual cortical sources (Halgren et al., 2002; Osterhout et al., 2004; Lau et al., 2008). Several methodological tools have been developed to overcome this mismatch. These include the recording of EEG signal from a larger number of electrodes, enabling source reconstruction of the signal through multiple dipole modeling (Darvas et al., 2006), or 3D registration of EEG signal onto high-resolution MRI images (Yoo et al., 1997). The latter method has already been explored in research combining EEG and fNIRS signals acquired simultaneously (Aihara et al., 2012; Morioka et al., 2014), with significant improvements in decoding accuracy (see Feature Extraction and Integration for more detail). Crucially, the possibility to incorporate source reconstruction methods into bimodal EEG-fNIRS research is contingent on the specific goals and constraints of individual studies. They may turn out particularly difficult to implement in studies seeking to minimize the number of sensors for wearability or convenience (e.g., in BCI technologies). Progress in developing source reconstruction from low-density EEG coverage (e.g., Guevara et al., 2020) or using anatomical priors from the fNIRS signal (Aihara et al., 2012), see also Feature Extraction and Integration) may provide a partial solution to these constraints and help further increase precision in the correspondence between fNIRS and EEG source localization.

Most studies in the review made use of custom-built EEG-fNIRS sensor setups from separate EEG and fNIRS equipment. However, such setups have been shown to create higher electrical interferences or noise in the EEG signal due to inadequate shielding of NIRS optode circuits and suboptimal return current paths (von Luhmann and Muller, 2017). Additionally, these setups typically involve using separate EEG and fNIRS event files that need to be fused after data acquisition—a procedure likely to alter synchronicity between them and therefore affect precision in event-timing reconstruction (von Luhmann and Muller, 2017). Built-in hybrid EEG-fNIRS systems on the other hand significantly reduce both issues (von Luhmann and Muller, 2017). Several such systems are now commercially available (example systems include Medelopt© by Seenel Imaging [cf. Safaie et al., 2013 and https://seenel-imaging.com/], Brite© by Artinis Medical Systems [cf. https://www.artinis.com/], g.Nautilus© by Gtec [cf. https://www.gtec.at] or NIRScout by NIRx [cf. https://nirx.net/], see also von Lühmann et al., 2017 and Kassab et al., 2018) and offer miniaturized, wireless modular technology allowing for greater mobility and flexibility of use across a wide range of real-life situations.

#### Feature Extraction and Integration

Bimodal EEG-fNIRS provides substantial amounts of complementary information that can be exploited by advanced signal processing algorithms for mental state classification. A precondition for accurate mental state decoding is the choice and appropriate extraction and integration of EEG and fNIRS features (Hong et al., 2018). Feature extraction implies transforming the raw signal into numerical information (i.e., “features”) that can be processed while preserving the essential information of the original data set. This information can be obtained from the time domain (mean, standard deviation, entropy, etc.), frequency domain (Fourier transform, wavelets, time-frequency distributions, etc.), or synchronicity between two or more spatial channels (coherence, correlation, mutual information, etc.). Naturally, efficient mental state decoding significantly depends on the proper methods of EEG and/or fNIRS feature extractions and classification. This particular aspect is the one in which the studies reviewed here vary the most, revealing the widespread selection of procedures available for achieving this crucial step in mental state decoding using bimodal EEG-fNIRS methodologies. Since no consensus exists as yet about which of these procedures are to be recommended, this point will be devoted to describing them in a way that highlights their primary distinctive characteristics.

*EEG features*. The most common EEG features selected for classification are the four major EEG frequency bands—i.e., delta (< 3 Hz), theta (3–8 Hz), alpha (and its motor homolog mu rhythm, 8–13Hz), and beta frequency (>13 Hz)—and their various subcategories (e.g., low/high beta (Abtahi et al., 2020), low/high alpha (Cicalese et al., 2020). Considerable research has been aimed at characterizing the physiological/functional significance of each of these frequency bands across a broad variety of tasks (Gevins and Smith, 2006), providing a wealth of prior knowledge to target appropriate task-specific frequencies for classification. Many of the studies reviewed therefore selected their frequency bands of interest based on a priori knowledge of their task relevance. Eight studies of motor execution and motor imagery for example focused on event-related synchronization/desynchronization patterns (ERS/ERD) characterized by short-time local alpha/mu suppression prior to movement onset followed by beta power increases after movement execution (Pfurtscheller et al., 1996; Pfurtscheller and Lopes da Silva, 1999). The functional significance of ERS/ERD-type patterns in motor execution and motor imagery has long been recognized within BCI research (McFarland et al., 2000), but ERD/ERS patterns also constitute reliable indices of high-level cognitive processing (Pfurtscheller and Lopes da Silva, 1999; Friedrich et al., 2013). Most studies investigating navigation (driving or flying) under well-rested vs. sleep-deprived conditions selected their frequency bands of interest based on previous literature or top-down analyses showing that lower frequencies (beta, alpha, and theta) reliably discriminated between well-rested vs. sleep-deprived conditions (Ahn et al., 2016; Nguyen et al., 2017; Dehais et al., 2019). Another study focused on decoding mental stress during task performance selected alpha and beta frequencies based on previous research showing that these frequency bands are reliable indicators of mental stress and cognitive engagement (Al-Shargie et al., 2016).

Other EEG features used, though less extensively than oscillatory frequencies, included wavelet approximation coefficients for motor event identification (Zhu et al., 2017; Sereshkeh et al., 2019), current density for EEG source reconstruction (Ge et al., 2019) or perception- and decision-specific ERP waveforms (Güven et al., 2020; e.g., N100, P200, P300, see Putze et al., 2014). One study (Fu et al., 2020) combined instantaneous amplitude, instantaneous phase, and instantaneous frequency of the EEG signal into a single vector in order to maximize classification performance, though little justification is given as to why integrating these particular features should enhance classification. Two clinically oriented studies selected their relevant EEG features based on their association with the neurological conditions of interest (i.e., interictal EEG discharge in epilepsy (Sirpal et al., 2019) and attention-related P300 in ADHD (Güven et al., 2020), achieving greater decoding accuracy for bimodal EEG-fNIRS compared to unimodal EEG and fNIRS. This illustrates the relevance of also exploiting abnormal EEG signatures in bimodal EEG-fNIRS decoding studies with diagnostic purposes.

##### fNIRS Features

fNIRS signal reflects variations in the concentration of oxygenated hemoglobin (conventionally indicated HbO) and deoxygenated hemoglobin (HbR, Pan et al., 2019), these values usually being negatively correlated with each other (Cui et al., 2009; Guerrero-Mosquera et al., 2016). HbO and HbR values together enable the calculation of the total hemoglobin mobilized during task performance (HbT). The studies reviewed here used these three values either alone or in combination. A majority of studies used HbO and HbR in combination. Eleven studies only used HbO and three used a combination of HbO, HbR, and HbT. Of the remaining studies, one used the difference between HbO and HbR as a relevant feature of interest (HbD). Since HbO and HbR typically show negative correlations with each other, its authors reasoned that HbD would further increase the amplitude of concentration changes as a function of task. This feature used in combination with HbO and HbR significantly improved classification accuracy in bimodal EEG-fNIRS. Another study computed the Hurst coefficient of HbO (Ge et al., 2017). The Hurst coefficient has been shown as a reliable measure of the internal consistency of time series produced by biological systems (Vorobyov and Cichocki, 2002). The authors however do not explain how this measure would improve the classification accuracy of the fNIRS signal. More generally, and contrary to EEG feature selection, little justification is explicitly provided across studies for choosing one fNIRS feature over others. In some cases (Morioka et al., 2014; Güven et al., 2020), the selection is based on prior evidence for the higher sensitivity of HbO compared to other fNIRS features in detecting cerebral blood flow (Hoshi et al., 2001). Other research using fMRI and NIRS however showed that the BOLD response as measured with fMRI was more correlated with HbR as measured by fNIRS (Huppert et al., 2006). In the case of studies with clinical populations (e.g., ADHD, cf. Morioka et al., 2014), the choice of HbO is in part motivated by prior evidence highlighting the suitability of this particular feature in detecting certain neurological conditions. Alternatively, other indicators of hemodynamic activity can be derived from recorded HbO and HbR such as cerebral oxygen exchange (COE) and cerebral blood volume vector (CBV) (Tanaka et al., 2014; Oka et al., 2015; Borragán et al., 2019). COE can show better sensitivity to detect changes in brain activity than HbO alone and is less affected by changes in CBV (Oka et al., 2015). The amplitude of low-frequency fluctuations (ALFF) is computed by assessing the oscillations within the frequency range 0.01-0.1 Hz and represents a good measure of local spontaneous activity. This measure has shown good performance to identify processes related to resting states or rumination (Lu et al., 2010; Rosenbaum et al., 2020).

##### EEG-fNIRS Integration

Efficient mental state decoding in bimodal EEG-fNIRS critically requires an appropriate combination of fNIRS and EEG features (Ahn and Jun, 2018; Li et al., 2019, 2020a,b). As explained in the introduction, however, fNIRS has substantially lower temporal resolution compared to EEG, a mismatch likely to severely limit the use of EEG-fNIRS in real-time studies typically requiring relatively high information transfer rates (Ahn and Jun, 2018). This temporal issue may also hinder proper source reconstruction of EEG signals based on the hemodynamic response provided by fNIRS. Several studies have addressed this issue by proposing a hierarchical Bayesian approach incorporating fNIRS and EEG (Aihara et al., 2012). In particular, rather than estimating source localization from electrophysiological current alone through current dipoles or distributed currents methods, both of which are insufficient for resolving the limited spatial resolution of EEG, the Bayesian approach imposes functional imaging data as a hierarchical prior to constrain estimation of the EEG current sources. This additional information has been shown to significantly help current source localization even with a small number of electrodes (Aihara et al., 2012). This approach was tested in the context of bimodal EEG-fNIRS research by Morioka et al. (2014) who compared its mental state decoding accuracy against an approach using EEG alone. Results showed that using fNIRS as a prior significantly increased decoding accuracy (79.1%) compared to using EEG alone (71.4%). Complementing these findings, Morioka et al. compared decoding performance between the Bayesian approach and another method of source reconstruction that did not use a hemodynamic signal as a prior (i.e., minimum L2-norm, cf. Wang et al., 1992). Results confirmed higher decoding accuracy for the hierarchical Bayesian approach (79.1 vs. 75.9%). Another potential avenue of progress lies in the detection of the so-called ‘initial dip' in the fNIRS signal, characterized by a local decrease in the HbO response around 1 second post-event onset and prior to the conventional increase in HbO (Zafar et al., 2016; Hong and Zafar, 2018). Improved techniques for detecting this initial dip have been considered among the methods for enhancing mental state decoding in BCI systems (Hong and Zafar, 2018). The study by Li et al. (2017) explored this issue in a bimodal EEG-fNIRS experiment on motor control in which fNIRS was used for initial dip detection. Improvement in decoding accuracy was significantly higher for bimodal EEG-fNIRS (91.02%) compared with unimodal EEG or fNIRS (< 86%). Finally, very recent studies have explored the possibility to use fluctuations in specific EEG frequency bands (e.g., gamma, alpha, or beta) to predict fluctuations in local fNIRS signal (Li et al., 2020a,b). Future research is expected to test which of these techniques might best improve decoding accuracy through more precise localization of task-related cortical activity.

#### EEG-fNIRS Classification and Detection Methods

Immediately following EEG and fNIRS feature extraction and integration is the actual processing of this integrated signal for accurate mental state decoding (cf. Figure 1). This decoding capacity largely depends on machine learning algorithms applied to brain imaging data (Lemm et al., 2011; Hong et al., 2018). Machine learning methods, including supervised/unsupervised learning algorithms and reinforcement learning, have now reached unprecedented levels of complexity in task outcomes such as classification, detection, or regression. These levels were achieved through increased computational power, efficient learning algorithms, valuable activation functions, and restricted or back-fed neuron connections (Pinti et al., 2015). The formal principles underlying the decoding of brain imaging data are detailed in Lemm et al. (2011). The following paragraphs provide an overview of the main decoding algorithms used in the studies reviewed here, assessing to the extent possible their relative merits and potential challenges.

The most common mental state classification technique used in the studies reviewed was the Linear Discriminant Analysis (LDA, 14 studies). LDA is a supervised algorithm that consists in breaking datasets down into two or more classes by maximizing the distance between the means of these classes while minimizing the variance within each class. This method's success lies in its low computational costs, making it suitable for use in online BCI systems. Second come Support Vector Machines (SVM, 11 studies), which work by first training a model in assigning data points to one or another category (supervised), ensuring that the distance between them and the class boundary is maximal, then using this trained model for assigning new incoming data to one or the other category. This method's primary strength is to enable nonlinear classification through the use of kernel functions (Hong et al., 2018). Seven studies used neural network algorithms for classification, including multilayered or Deep Neural Networks (DNNs), (Cichy and Kaiser, 2019). A variant of simple neural networks aimed at simulating learning as instantiated in the human brain, DNNs feature several (more than two) interconnected layers of processing units (i.e. neurons) which increase their reciprocal connections as a function of learning. These algorithms' principal specificity is to directly use non-preprocessed data for classification (Schirrmeister et al., 2017; Saadati et al., 2020), feeding it into varying numbers of convolutional layers enabling them to decode the signal at different levels of specificity and abstraction. Their primary advantage compared to LDA or SVM is to bypass a priori feature selection and automatically detect hierarchical patterns of information from the raw signal. This improvement however comes with the potential drawback of yielding wrong learning outputs or requiring longer periods of learning (Schirrmeister et al., 2017, but see Yin et al., 2015). Saadati et al. (2020) directly compared classification accuracies between SVM and a deep neural network (DNN) from bimodal EEG-fNIRS signals recorded in a number of cognitive and motor tasks. Not only did EEG-fNIRS perform better than unimodal EEG or fNIRS, but classification accuracy was overall higher for DNN (~90%) than for SVM (~84%). Another study aimed at discriminating children with ADHD vs. control during an ‘oddball' detection paradigm (Güven et al., 2020) directly compared discrimination accuracies between three supervised algorithms using EEG, fNIRS, and EEG-fNIRS features: SVM, the multilayer perception network and the naïve Bayes classifier. Results showed that accuracy was highest for EEG-fNIRS but did not differ between SVM and the neural network (86.3%). The highest accuracy level was obtained through the use of the naïve Bayes classifier (93.2%).

New methods of mental state decoding from bimodal EEG-fNIRS signals are still being developed. Most recent work in this respect has focused on optimizing procedures for fusing EEG and fNIRS signals, with important improvements in decoding accuracy compared to previous classification algorithms (Khan and Hasan, 2020; Sun et al., 2020). Also, the spatial relationship between EEG and fNIRS signals have been used to feed up deep learning architectures for classification, such as convolutional neural networks (RCNNs) for mental task (precision of 99.6%, cf. Ghonchi et al., 2020) or CNNs for mental workload (accuracy 89%, cf. Saadati et al., 2020). Furthermore, with the rapid development of BCI technology, the feasibility of new hardware setups using few EEG channels and fNIRS source-detector pairs has been tested in three different mental tasks using shrinkage LDA classifier), and also for channel selection through k-nearest neighbor (kNNs) and Tree classifiers (Hasan et al., 2020). These results show classification accuracy high enough to be used in practical BCI applications.

### EEG-fNIRS Use in Ecologically Valid Contexts

As mentioned at the outset the enhanced wearability of bimodal EEG and fNIRS instrumentation should in principle facilitate the monitoring of brain activity in real-world situations. It must be noted however that almost all the studies reviewed here were still performed in laboratory settings, highlighting the many remaining challenges of using bimodal EEG-fNIRS equipment in ecologically valid contexts. Further progress in this domain depends in part on the availability of miniaturized hybrid EEG-fNIRS setups and signal (pre-)processing algorithms handling motion-related artifacts in a near-instantaneous fashion. Another challenge comes from the versatility of real-world over laboratory conditions where events of interest can be precisely identified through computer-controlled experimental scenarios (Pinti et al., 2017; von Lühmann et al., 2021). An important short-term objective is to be able to retrace the history of an experiment in the absence of scenario files. While little if any progress has been achieved in the particular case of bimodal EEG-fNIRS several methods have been developed for unimodal fNIRS that could be extended to bimodal EEG-fNIRS research. Pinti et al. (2015) for example made use of video cameras fitted onto subjects and accompanying experimenters. Careful analysis of video recordings allowed authors to recover functional events of interest and to use them for tolerably good HbO and HbR measurements in two separate conditions. Another approach developed by the same authors consists in recovering functional events in the fNIRS signal through a GLM-based data-driven method (Pinti et al., 2017). Used in combination with the video recordings on the same 2015 study dataset, this method allowed authors to recover 50 to 75 percent of functionally relevant events. Crucially, however, these methods remain limited by the temporal precision of fNIRS, which is not fitted to capture the versatility and rapidity of real-world environments the way EEG is (Casson, 2019). In this respect, recent research has concentrated on building wearable EEG setups capable of retracing functional events through eye-fixation data (Casson and Trimble, 2018). Future efforts should be geared at combining these methods.

## Recommendations For Future Neuroergonomics Research Using EEG-fNIRS

The main focus of neuroergonomics is to build human-machine interfaces for assisting safe and autonomous real-world performance in populations with motor or communicative disabilities, or healthy individuals engaged in high-risk private or professional activities. The recent emergence of bimodal EEG-fNIRS systems apt to decode performance-related mental states as part of these interfaces has received increased attention. This review article aimed to examine the level of improvement achieved in mental state decoding using bimodal EEG-fNIRS compared to unimodal EEG or fNIRS. It also considered the state of progress and challenges in implementing wearable versions of these interfaces. Its results reveal consistent increases in decoding accuracy for bimodal over unimodal EEG and fNIRS despite a limited number of studies and significant variation in the studies' research questions and methods. They also highlight several important challenges in the real-world use of bimodal EEG-fNIRS systems. The remaining paragraphs synthesize these points in the form of recommendations for future research.

First, increased effort should be placed on achieving higher consistency in terms of sensor setups, feature selection, and extraction methods. In terms of sensor setup hybrid EEG-fNIRS hardware should be favored over custom EEG-fNIRS systems built from separate fNIRS and EEG equipment to guard against potential crosstalk and event-timing offsets between EEG and fNIRS signals. Additionally, sensor layouts should be designed by taking into consideration the discrepancies between EEG and fNIRS regarding temporal vs. spatial granularity. Compared with the relatively unambiguous spatial distribution of fNIRS responses the scalp distribution of EEG signatures can be misleading in terms of signal sources. Depending on the level of sophistication in the experimental paradigm and sensor layout, it should therefore be ensured that the fNIRS and EEG signal captured in the same scalp area are tied to the same underlying processes. Conversely, the greater spatial resolution of fNIRS compared to EEG comes at the cost of larger temporal latencies likely to cause difficulties in the real-time monitoring of performance-related brain-signal. Though several avenues have been explored to minimize the temporal discrepancy between EEG and fNIRS, it remains difficult if not impossible to align both modalities at the same level of temporal resolution. Future research should hopefully help minimize or compensate for these temporal discrepancies.

Second, as decoding accuracy naturally depends on signal quality the relevance of signal preprocessing and artifact rejection methods cannot be overlooked. These methods should take into account both modality-specific and modality-independent artifacts, appropriately distinguish between systemic and nonsystemic sources of noise and account for the constraints associated with signal preprocessing such as stimulus repetition and averaging. The latter point poses a particular challenge in the attempt to decode mental states in real-world contexts devoid of repetition and regularity. It is therefore central to focus on the development of online preprocessing algorithms and systems that can optimize noise rejection on single events.

Third, increased effort should also be put into a more targeted and justified selection and integration of EEG and fNIRS features. In the case of EEG, the most common features are the major brain oscillatory frequencies, while the use of event-related potentials remains relatively marginal. In most cases, the frequency bands of interest were selected based on prior knowledge of their functional involvement in the target tasks. In the case of fNIRS, the main features of interest included local changes in HbO, HbR, and HbT. Compared to EEG features, however, little justification is given for selecting one over the other fNIRS features. Possible reasons for this gap include limited knowledge of the relation between hemodynamic signal and underlying neurocognitive processes and of the functional significance of variations in local oxy- or deoxyhemoglobin concentration. Available evidence indicates a correspondence between HbR and HbT concentration and negative bold responses as measured through fMRI (Maggioni et al., 2015). The exact meaning of negative blood dynamics however has remained elusive (Wade, 2002).

Fourth, whereas an immediate goal of neuroergonomics research is to decode task-related mental states in naturalistic conditions, most studies featured in the review were still performed in highly controlled laboratory settings. This indicates that the multimodal exploitation of EEG-fNIRS in real-life applications is still in its infancy. The most likely conditions to meet in achieving this goal include proper miniaturization of EEG-fNIRS systems, the development of advanced algorithms for signal preprocessing, and more powerful methods of real-time EEG and fNIRS analysis. Another major challenge concerns the precise tracking of a sufficient number of events of interest. Overcoming these challenges should not only pave the way toward a better understanding of the neurocognitive correlates of human performance but should open new possibilities for equipping modern hardware (e.g., vehicles, prosthetic devices, etc.) apt to usefully exploit mental states for assisting or enhancing behaviors. On this particular point, it is worth acknowledging the possibility to complement EEG-fNIRS signal with other behavioral and physiological measures. These include motion capture, heart rate, skin conductance, or eye-tracking technologies. The studies reviewed that exploited these additional measures report further improvements in decoding accuracy.

Finally, although decoding is shown to be better in the bimodal than the unimodal approach, this observation is for many studies based on absolute percentage values rather than formal statistical comparisons. Until such comparisons are systematically made the merits of bimodal EEG-fNIRS over unimodal EEG or fNIRS in mental state decoding should therefore be considered with caution. It must be remarked how few studies reviewed approach their research question from a theoretical vantage. Regardless of the issue investigated, little conceptual work is exploited to predict or account for the patterns reported. This point might seem unfortunate given the considerable potential of bimodal EEG-fNIRS in moving neuroergonomics research forward at both fundamental and practical levels (Pfurtscheller et al., 2012; Wallois et al., 2012; Balconi et al., 2015; Pinti et al., 2018b). The development of advanced tools for investigating the neurocognitive correlates of behavior does not replace careful theoretical analysis of the phenomenon addressed (Krakauer et al., 2017). A strong working definition of the cognitive processes targeted by specific experiments is of fundamental importance in an effort to decode mental states from electrophysiological or hemodynamic signal.

## Conclusion

The advent of portable methods of multimodal brain monitoring foreshadows significant progress in understanding and exploiting performance-related mental states in naturalistic situations. Amongst these methods, bimodal EEG-fNIRS technologies potentially represent an important step forward in this direction. Available evidence, though recent and relatively limited in scope and amount, consistently points to significant improvements in the decoding of performance-related mental states, with crucial implications in both the conceptual and practical domains of NE. Many challenges however still stand in the way of fully exploiting these technologies in the service of human factors research and development. Relevant questions to be addressed in future research in our view comprise the following: First, is bimodal EEG-fNIRS systematically more desirable than unimodal EEG or fNIRS across all domains of NE? The superior decoding accuracy of bimodal EEG-fNIRS would intuitively seem to bring a positive answer to this question, but one must also weigh the gains of combining these technologies in human-machine interface systems against the costs incurred by their limitations. It is possible in practice that unimodal technologies remain at present more efficient due to their greater flexibility of use in real-world situations as well as a higher degree of technical and conceptual knowledge of their neuroergonomic potential. Most recent state-of-the-art reports highlight the considerable technological advances afforded by portable EEG in neuroergonomics research (Wascher et al., 2021). In contrast, neuroergonomic applications of fNIRS still exhibit comparably greater conceptual and methodological shortcomings (Zhu et al., 2020). Only when these gaps are overcome will it be possible to appreciate the scope and limits of multimodal brain imaging in human-machine interface applications.

Second, do the improvements afforded by bimodal EEG-fNIRS alleviate the need for theoretically grounded approaches to their exploitation in the neuroergonomic context? As has been noted above few of the studies reviewed ground their research into fine-grained theoretical considerations of human performance and its underlying cognitive processes as reflected in the EEG and fNIRS signal. This theory-free approach might in part be symptomatic of the general enthusiasm for the ever-increasing multiplication and sophistication of novel techniques of neuroscientific investigation, which may in turn overshadow the need for deep conceptual analyses of how behavior emerges from the computational operations of the brain (Krakauer et al., 2017). Such analyses however remain in our view an essential precondition for improvement in the use of multimodal brain imaging in human-machine interface systems, particularly with regard to the choice of appropriate decoding algorithms (Kriegeskorte and Douglas, 2018).

## Author Contributions

NB, SL, GB, and CG-M conceived the review and wrote the paper. NB performed the review. All authors contributed to the article and approved the submitted version.

## Funding

This work was supported by the Belgian Royal Higher Institute for Defense (Grant HFM20-02 awarded to SL).

## Conflict of Interest

The authors declare that the research was conducted in the absence of any commercial or financial relationships that could be construed as a potential conflict of interest.

## Publisher's Note

All claims expressed in this article are solely those of the authors and do not necessarily represent those of their affiliated organizations, or those of the publisher, the editors and the reviewers. Any product that may be evaluated in this article, or claim that may be made by its manufacturer, is not guaranteed or endorsed by the publisher.
