# Supplementary material for: Bimodal EEG-fNIRS in Neuroergonomics. Current Evidence and Prospects for Future Research
Source: Front Neuroergon. 2022 Aug 12;3:934234. doi: 10.3389/fnrgo.2022.934234 (PMC10790898; doi:10.3389/fnrgo.2022.934234)
Supplement: Supplementary file 1 [file Data_Sheet_1.docx]

**Supplementary Document 1**

**Explanation of Existing Methods for AAL Implementation**

**Implementation 1 (Piecewise product of probability and loss):**

**Table 1.** Flood AAL by first-floor height.

|  | **Flood Loss (USACE 2000 DDF)** | | | |  |
| --- | --- | --- | --- | --- | --- |
| **FFH** | **10-year** | **50-year** | **100-year** | **500-year** | **AAL (Equation 1)** |
| 3.0 | 14,853 | 22,625 | 27,153 | 34,476 | $2,063 |
| 3.5 | 6,798 | 14,853 | 19,550 | 27,153 | $1279 |
| 4.0 | 0 | 6,798 | 11,665 | 19,550 | $528 |
| 4.5 | 0 | 0 | 3,496 | 11,665 | $101 |
| 5.0 | 0 | 0 | 0 | 3,496 | $21 |
| 5.5 | 0 | 0 | 0 | 0 | $0 |


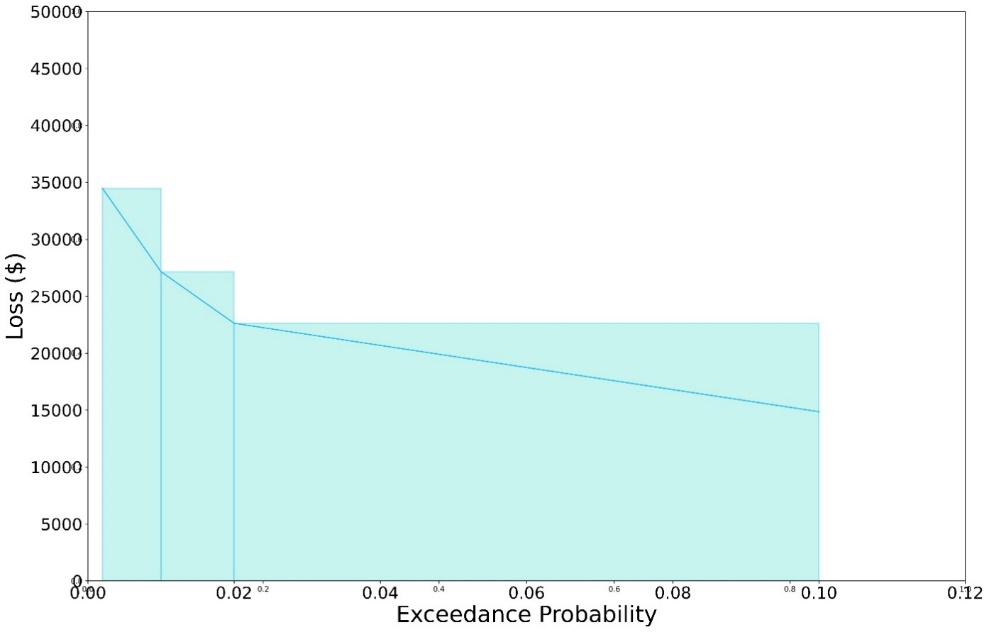


**Figure 1.** Loss-Exceedance probability curve for FFH = 3.0.

**Implementation 2 (Log-Linear method):**

**Case Study with USACE 2000 Building DDF**

Table 1 is used to estimate the slope and intercept from Equation 2 in the main text. The values are provided in Table 2.

(a) (b)

**Figure 2.** Loss-log of return period curve for (a) FFH = 3.0 (b) FFH = 3.5.

**Table 2.** Regression parameters and AAL as a function of first-floor height.

| **FFH** | **slope** | **intercept** | **AAL ($)** |
| --- | --- | --- | --- |
| 3.0 | 5062 | 3219 | 10,309 |
| 3.5 | 5251 | -5274 | 1,151 |
| 4.0 | 5059 | -12041 | 477 |
| 4.5 | 3045 | -9175 | 150 |
| 5.0 | 866 | -2816 | 33 |
| 5.5 | 0 | 0 | 0 |

**Figure 3.** Loss-log of return period curve for FFH = 3.

**Figure 4.** Loss-exceedance probability curve for FFH = 3.

**Implementation 3 (GEV):**

Script:

The R code is provided by the authors of Zarekarizi et al. (2020) paper: https://github.com/scrim-network/Zarekarizi-flood-home-elavate

**Case Study**

The gage height (feet) data of river gage station USGS 07374525 Mississippi river at Belle Chasse, LA was downloaded from USGS website.

Description of the Gage station: Latitude 29°51'25", Longitude 89°58'40" NAD27

Plaquemines Parish, Louisiana, Hydrologic Unit 08090100

Drainage area: 1,130,000 square miles

Contributing drainage area: 1,110,000 square miles,

Datum of gage: -6.58 feet above NAVD88.

The data were processed and used to fit a Generalized Extreme Value distribution (GEV). The “S10_Estimate_MCMC.R” script was used to estimate the GEV parameters.

GEV parameters:

mu = 17.2137

sigma = 1.2270

xi = -1.2829

Then, “EAD_Function.R” script was used to estimate the average annual losses.
